# Scoping studies: advancing the methodology

**DOI:** 10.1186/1748-5908-5-69

**Published:** 2010-09-20

**Authors:** Danielle Levac, Heather Colquhoun, Kelly K O'Brien

**Affiliations:** 1 School of Rehabilitation Science, McMaster University, 1400 Main Street West, Room 403, Hamilton, Ontario, Canada; 2 Department of Physical Therapy, University of Toronto, 160-500 University Avenue, Toronto, Ontario, Canada

## Abstract

**Background:**

Scoping studies are an increasingly popular approach to reviewing health research evidence. In 2005, Arksey and O'Malley published the first methodological framework for conducting scoping studies. While this framework provides an excellent foundation for scoping study methodology, further clarifying and enhancing this framework will help support the consistency with which authors undertake and report scoping studies and may encourage researchers and clinicians to engage in this process.

**Discussion:**

We build upon our experiences conducting three scoping studies using the Arksey and O'Malley methodology to propose recommendations that clarify and enhance each stage of the framework. Recommendations include: clarifying and linking the purpose and research question (stage one); balancing feasibility with breadth and comprehensiveness of the scoping process (stage two); using an iterative team approach to selecting studies (stage three) and extracting data (stage four); incorporating a numerical summary and qualitative thematic analysis, reporting results, and considering the implications of study findings to policy, practice, or research (stage five); and incorporating consultation with stakeholders as a required knowledge translation component of scoping study methodology (stage six). Lastly, we propose additional considerations for scoping study methodology in order to support the advancement, application and relevance of scoping studies in health research.

**Summary:**

Specific recommendations to clarify and enhance this methodology are outlined for each stage of the Arksey and O'Malley framework. Continued debate and development about scoping study methodology will help to maximize the usefulness and rigor of scoping study findings within healthcare research and practice.

## Background

Scoping studies (or scoping reviews) represent an increasingly popular approach to reviewing health research evidence [1]. However, no universal scoping study definition or purpose exists (Table 1) [1,2]. Definitions commonly refer to 'mapping,' a process of summarizing a range of evidence in order to convey the breadth and depth of a field. Scoping studies differ from systematic reviews because authors do not typically assess the quality of included studies [3-5]. Scoping studies also differ from narrative or literature reviews in that the scoping process requires analytical reinterpretation of the literature [1].

**Table 1:** Definitions and purposes of scoping studies

Authors	Definition	Purpose(s)
**Ehrich *et al. *(2002)**	None provided.	'The purpose of a scoping exercise is both to map a wide range of literature, and to envisage where gaps and innovative approaches may lie"' [[11] p. 28].

**Arksey and O'Malley (2005)**	'Aim to map rapidly the key concepts underpinning a research area and the main sources and types of evidence available' [[14] p. 194], as cited in [6]	1. To examine the extent, range, and nature of research activity. 2. To determine the value for undertaking a full systematic review. 3. To summarize and disseminate research findings. 4. To identify research gaps in the existing literature. [[6] p. 21]

**Anderson *et al. *(2008)**	'Scoping studies are concerned with contextualizing knowledge in terms of identifying the current state of understanding; identifying the sorts of things we know and do not know; and then setting this within policy and practice contexts' [2]	1. Literature mapping: 'is a map of the relevant literature. These vary in scope from general accounts of the literature to studies that are just short of systematic reviews. Literature scoping studies often also involve the syntheses of findings from different types of study.' 2. Conceptual mapping: 'a scoping study designed to establish how a particular term is used in what literature, by whom and for what purpose.' 3. Policy mapping: 'a scoping study designed to identify the main documents and statements from government agencies and professional bodies that have a bearing on the nature of practice in that area.'' 4. Stakeholder consultation: 'Do[es] not constitute scoping studies in their own right, but they do have an important part to play in scoping studies concerned with the identification of research priorities, in helping to target research questions, and in validating the outcomes of scoping studies through peer-review' [2].

**Grant *et al. *(2009)**	'Preliminary assessment of potential size and scope of research literature.' [[4] p.95]	'Aims to identify the nature and extent of research evidence (usually including ongoing research' [[4] p.95].

**Davis *et al. *(2009)**	'Scoping involves the synthesis and analysis of a wide range of research and non-research material to provide greater conceptual clarity about a specific topic or field of evidence' [[1] p.1386].	'We propose that a common synthesising construct emerges to explain the purpose of scoping, namely that of 'reconnaissance'. It is generally synonymous with a preliminarily investigation in which information is systematically gathered and examined in order to establish strengths and weakness and guide in which ever context, future decision-making' [[1] p. 1396].

**National Institute for** **Health Research (NIHR) Service Delivery and Organisation** **Research and Development Programme (SDO)**	None provided.	1.'Clarification of working definitions and conceptual boundaries of a topic area, developed in the form of systematic overview (narrative review) of the literature but specifically excluding a systematic review, to determine a frame of reference; 2. Outline what is already known and identify gaps in existing research, and; 4. Conceptual analysis may include the 'mapping' of existing empirical evidence to describe and interpret issues that will inform further research and development opportunities.' [[1] p. 1387]

**Canadian Institutes of Health Research** **http://www.cihr-irsc.ca**	'Scoping reviews are exploratory projects that systematically map the literature available on a topic, identifying the key concepts, theories, sources of evidence, and gaps in the research. They are often preliminary to full syntheses, undertaken when feasibility is a concern -- either because the potentially relevant literature is thought to be especially vast and diverse (varying by method, theoretical orientation or discipline) or there is suspicion that not enough literature exists. These entail the systematic selection, collection and summarization of existing knowledge in a broad thematic area for the purpose of identifying where there is sufficient evidence to conduct a full synthesis or where insufficient evidence exists and further primary research is necessary.' [15]	None provided.

Researchers can undertake a scoping study to examine the extent, range, and nature of research activity, determine the value of undertaking a full systematic review, summarize and disseminate research findings, or identify gaps in the existing literature [6]. As such, researchers can use scoping studies to clarify a complex concept and refine subsequent research inquiries [1]. Scoping studies may be particularly relevant to disciplines with emerging evidence, such as rehabilitation science, in which the paucity of randomized controlled trials makes it difficult for researchers to undertake systematic reviews. In these situations, scoping studies are ideal because researchers can incorporate a range of study designs in both published and grey literature, address questions beyond those related to intervention effectiveness, and generate findings that can complement the findings of clinical trials.

In an effort to provide guidance to authors undertaking scoping studies, Arksey and O'Malley [6] developed a six-stage methodological framework: identifying the research question, searching for relevant studies, selecting studies, charting the data, collating, summarizing, and reporting the results, and consulting with stakeholders to inform or validate study findings (Table 2). While this framework provided an excellent methodological foundation, published scoping studies continue to lack sufficient methodological description or detail about the data analysis process, making it challenging for readers to understand how study findings were determined [1]. Arksey and O'Malley [6] encouraged other authors to refine their framework in order to enhance the methodology.

**Table 2:** Overview of the Arksey and O'Malley methodological framework for conducting a scoping study

Arksey and O'Malley Framework Stage	Description
1: Identifying the research question	Identifying the research question provides the roadmap for subsequent stages. Relevant aspects of the question must be clearly defined as they have ramifications for search strategies. Research questions are broad in nature as they seek to provide breadth of coverage.

2: Identifying relevant studies	This stage involves identifying the relevant studies and developing a decision plan for where to search, which terms to use, which sources are to be searched, time span, and language. Comprehensiveness and breadth is important in the search. Sources include electronic databases, reference lists, hand searching of key journals, and organizations and conferences. Breadth is important; however, practicalities of the search are as well. Time, budget and personnel resources are potential limiting factors and decisions need to be made upfront about how these will impact the search.

3: Study selection	Study selection involves *post hoc *inclusion and exclusion criteria. These criteria are based on the specifics of the research question and on new familiarity with the subject matter through reading the studies.

4: Charting the data	A data-charting form is developed and used to extract data from each study. A 'narrative review' or 'descriptive analytical' method is used to extract contextual or process oriented information from each study.

5: Collating, summarizing, and reporting results	An analytic framework or thematic construction is used to provide an overview of the breadth of the literature but not a synthesis. A numerical analysis of the extent and nature of studies using tables and charts is presented. A thematic analysis is then presented. Clarity and consistency are required when reporting results.

6: Consultation (optional)	Provides opportunities for consumer and stakeholder involvement to suggest additional references and provide insights beyond those in the literature.

In this paper, we apply our experiences using the Arksey and O'Malley framework to build on the existing methodological framework. Specifically, we propose recommendations for each stage of the framework, followed by considerations for the advancement, application, and relevance of scoping studies in health research. Continual refinement of the framework stages may provide greater clarity about scoping study methodology, encourage researchers and clinicians to engage in this process, and help to enhance the methodological rigor with which authors undertake and report scoping studies [1].

## Discussion

We each completed a scoping study in separate areas of rehabilitation using the Arksey and O'Malley framework [6]. Goals of these studies included: identifying research priorities within HIV and rehabilitation [7], applying motor learning strategies within pediatric physical and occupational therapy intervention approaches [8], and exploring the use of theory within studies of knowledge translation [9]. The amount of literature reviewed in our studies ranged from 31 (DL) to 146 (KO) publications. Upon discovering that we had similar challenges implementing the scoping study methodology, we decided to use our experiences to further develop the existing framework. We conducted an informal literature search on scoping study methodology. We searched CINAHL, MEDLINE, PubMed, ERIC, PsycInfo, and Web of Science databases using the search terms 'scoping,' 'scoping study,' 'scoping review,' and 'scoping methodology' for papers published in English between January 1990 and May 2010. Reference lists of pertinent papers were also searched. This search yielded seven citations that reflected on scoping study methodology, which were reviewed by one author (DL). After independently considering our own experiences utilizing the Arskey and O'Malley [6] framework, we met on seven occasions to discuss the challenges and develop recommendations for each stage of the methodological framework.

### Recommendations to enhance scoping study methodology

We outline the challenges and recommendations associated with each stage of the methodological framework (Table 3).

**Table 3:** Summary of challenges and recommendations for scoping studies

Framework Stage	Challenges	Recommendations for clarification or additional steps
#1 Identifying the research question	1. Scoping study questions are broad. 2. Establishing scoping study purpose is not associated with a framework stage. 3. The four purposes of scoping studies lack clarity.	1. Clearly articulate the research question that will guide the scope of inquiry. Consider the concept, target population, and health outcomes of interest to clarify the focus of the scoping study and establish an effective search strategy. 2. Mutually consider the purpose of the scoping study with the research question. Envision the intended outcome (*e.g*., framework, list of recommendations) to help determine the purpose of the study. 3. Consider rationale for conducting the scoping study to help clarify the purpose.

#2 Identifying relevant studies	1. Balancing breadth and comprehensiveness of the scoping study with feasibility of resources can be challenging.	1a. Research question and purpose should guide decision-making around the scope of the study. 1b. Assemble a suitable team with content and methodological expertise that will ensure successful completion of the study. 1c. When limiting scope is unavoidable, justify decisions and acknowledge the potential limitations to the study.

#3 Study selection	1. The linearity of this stage is misleading. 2. The process of decision making for study selection is unclear.	1. This stage should be considered an iterative process involving searching the literature, refining the search strategy, and reviewing articles for study inclusion. 2a. At the beginning of the process, the team should meet to discuss decisions surrounding study inclusion and exclusion. At least two reviewers should independently review abstracts for inclusion. 2b. Reviewers should meet at the beginning, midpoint and final stages of the abstract review process to discuss challenges and uncertainties related to study selection and to go back and refine the search strategy if needed. 2c. Two researchers should independently review full articles for inclusion. 2d. When disagreements on study inclusion occur, a third reviewer can determine final inclusion.

#4 Charting the data	1. The nature and extent of data to extract from included studies is unclear. 2. The 'descriptive analytical method' of charting data is poorly defined.	1a. The research team should collectively develop the data-charting form and determine which variables to extract in order to answer the research question. 1b. Charting should be considered an iterative process in which researchers continually extract data and update the data-charting form. 1c. Two authors should independently extract data from the first five to ten included studies using the data-charting form and meet to determine whether their approach to data extraction is consistent with the research question and purpose. 2. Process-oriented data may require extra planning for analysis. A qualitative content analysis approach is suggested.

#5 Collating, summarizing, and reporting the results	1. Little detail provided and multiple steps are summarized as one framework stage.	Researchers should break this stage into three distinct steps: 1a. Analysis (including descriptive numerical summary analysis and qualitative thematic analysis); 1b. Reporting the results and producing the outcome that refers to the overall purpose or research question; 1c. Consider the meaning of the findings as they relate to the overall study purpose; discuss implications for future research, practice and policy.

#6 Consultation	1. This stage is optional. 2. Lack of clarity exists about when, how and why to consult with stakeholders and how to integrate the information with study findings.	1. Consultation should be an essential component of scoping study methodology. 2a. Clearly establish a purpose for the consultation. 2b. Preliminary findings can be used as a foundation to inform the consultation. 2c. Clearly articulate the type of stakeholders to consult and how data will be collected, analyzed, reported and integrated within the overall study outcome. 2d. Incorporate opportunities for knowledge transfer and exchange with stakeholders in the field.

### Framework stage one: Identifying the research question

Scoping study research questions are broad in nature as the focus is on summarizing breadth of evidence. Arksey and O'Malley [6] acknowledge the need to maintain a broad scope to research questions, however we found our research questions lacked the direction, clarity, and focus needed to inform subsequent stages of the research process, such as identifying studies and making decisions about study inclusion. To clarify this stage, we recommend that researchers combine a broad research question with a clearly articulated scope of inquiry. This includes defining the concept, target population, and health outcomes of interest to clarify the focus of the scoping study and establish an effective search strategy. For example, in one author's (KO) scoping study, the research question was broadly 'what is known about HIV and rehabilitation?' Defining the concept of 'rehabilitation' was essential in order to establish a clear scope to the study, guide the search strategy, and establish parameters around study selection in subsequent stages of the process [7].

Although Arskey and O'Malley [6] outline four main purposes for undertaking a scoping study, they do not articulate that purpose be specified within a specific framework stage. We recommend researchers simultaneously consider the purpose of the scoping study when articulating the research question. Linking a clear purpose for undertaking a scoping study to a well-defined research question at the first stage of the framework will help to provide a clear rationale for completing the study and facilitate decision making about study selection and data extraction later in the methodological process. A helpful strategy may be to envision the content and format of the intended outcome that may assist researchers to clearly determine the purpose at the beginning of a study. In the abovementioned HIV study, authors linked the broadly stated research question with a more specific purpose 'to identify the key research priorities in HIV and rehabilitation to advance policy and practice for people living with HIV in Canada' [7]. The envisioned outcome was a thematic framework that represented strengths and opportunities in HIV rehabilitation research, followed by a list of the key research priorities to pursue in future work.

Finally, the purposes put forth by Arksey and O'Malley [6] require more debate. We concur with Anderson *et al. *[2] and Davis *et al. *[1], who state that researchers may benefit from further clarification of the purposes for undertaking a scoping study. The first purpose, as articulated by Arksey and O'Malley [6], is to summarize the extent, range, and nature of research activity; however, researchers are not required to reflect on their underlying motivation for doing so. We recommend that researchers consider the rationale for why they should summarize the activity in a field and the implications that this will have on research, practice, or policy. The second purpose is to assess the need for a full systematic review. However, it is difficult to determine whether a systematic review is advantageous when a scoping study does not involve methodological quality assessment of included studies. Furthermore, it is unclear how this purpose differs from existing methods of determining feasibility for a systematic review. The third purpose is to summarize and disseminate research findings, but we question how this differs from other narrative or systematic literature reviews. Lastly, the fourth purpose of undertaking a scoping study -- to identify gaps in the existing literature -- may yield false conclusions about the nature and extent of those gaps if the quality of the evidence is not assessed. The purpose 'to identify the key research priorities in HIV and rehabilitation to advance policy and practice for people living with HIV in Canada' does not explicitly align with one of the four Arskey and O'Malley purposes [7]. However, it appears authors inherently first summarized the extent, range, and nature of research (purpose one) and identified gaps in the existing literature (purpose four) in order to subsequently identify the key research priorities in HIV and rehabilitation (author purpose). This suggests authors might have an overall study purpose with multiple objectives articulated by Arksey and O'Malley that are required in order to help achieve their overall purpose.

### Framework stage two: Identifying relevant studies

A strength of scoping studies includes the breadth and depth, or comprehensiveness, of evidence covered in a given field [1]. However, practical issues related to time, funding, and access to resources often require researchers to consider the balance between feasibility, breadth, and comprehensiveness. Brien *et al. *[5] reported that their search strategy yielded a vast amount of literature, making it difficult to determine how in depth to carry out the information synthesis. Although Arksey and O'Malley [6] identify these concerns and provide some suggestions to support these decisions, we also struggled with the trade-off between breadth and comprehensiveness and feasibility in our scoping studies. As such, we recommend that researchers ensure decisions surrounding feasibility do not compromise their ability to answer the research question or achieve the study purpose. Second, we recommend that a scoping study team be assembled whose members provide the methodological and context expertise needed for decisions regarding breadth and comprehensiveness. When limiting scope is unavoidable, researchers should justify their decisions and acknowledge the potential limitations of their study.

### Framework stage three: Study selection

Arksey and O'Malley [6] provide suggestions to manage the time-consuming process of determining which studies to include in a scoping study. We experienced this stage as more iterative and requiring additional steps than implied in the original framework. While Arksey and O'Malley [6] do not indicate a team approach is imperative, we agree with others and suggest scoping studies involve multidisciplinary teams using a transparent and replicable process [2,10]. In two of our studies (HC and DL) where decision making was primarily completed by a single author, we faced several challenges, including uncertainty about which studies to include, variables to extract on the data-charting form, and the nature and extent of detail to conduct the data extraction process. This raised questions related to rigor and led to our recommendations for undertaking a systematic team approach to conducting a scoping study.

Specifically, we recommend that the team meet to discuss decisions surrounding study inclusion and exclusion at the beginning of the scoping process. Refining the search strategy based on abstracts retrieved from the search and reviewing full articles for study inclusion is also a critical step. We recommend that at least two researchers each independently review abstracts yielded from the search strategy for study selection. Reviewers should meet at the beginning, midpoint, and final stages of the abstract review process to discuss any challenges or uncertainties related to study selection and to go back and refine the search strategy if needed. This can help to alleviate potential ambiguity with a broad research question and to ensure that abstracts selected are relevant for full article review. Next, two reviewers should independently review the full articles for inclusion. When disagreements occur, a third reviewer can be consulted to determine final inclusion.

### Framework stage four: Charting the data

This stage involves extracting data from included studies. Based on our experiences, we were uncertain about the nature and extent of information to extract from the included studies. To clarify this stage, we recommend that the research team collectively develop the data-charting form to determine which variables to extract that will help to answer the research question. Secondly, we recommend that charting be considered an iterative process in which researchers continually update the data-charting form. This is particularly true for process-oriented data, such as understanding how a theory or model has been used within a study. Uncertainty about the nature and extent of data that should be extracted may be resolved by researchers beginning the charting process and becoming familiar with study data, and then meeting again to refine the form. We recommend an additional step to charting the data in which two researchers independently extract data from the first five to ten studies using the data-charting form and meet to determine whether their approach to data extraction is consistent with the research question and purpose. Researchers may review one study several times within this stage. The number of researchers involved in the data extraction process will likely depend upon the number of included studies. For example, in one study, authors had difficulty developing one data-charting form that could apply to all included studies representing a range study designs, reviews, reports, and commentaries [7]. As a preliminary step, authors decided to classify the included studies into three areas --HIV disability, interventions, and roles of rehabilitation professionals in HIV care -- to help determine the nature and extent of information to extract from each of the types of studies [7].

Arksey and O'Malley [6] refer to a 'descriptive analytical method' that involves summarizing process information, such as the use of a theory or model in a meaningful format. Our experiences indicated that this is a highly valuable, though challenging aspect of scoping studies, as we struggled to chart and summarize complex concepts in a meaningful way. Arksey and O'Malley [6] indicate that synthesis of material is critical as scoping studies are not a short summary of many articles. We agree, and feel that additional direction in the framework might help to navigate this crucial but challenging stage. Perhaps synthesizing process information may benefit from utilization of qualitative content analysis approaches to make sense of the wealth of extracted data [11]. This issue also highlights the overlap with the next analytical stage. The role and relevance of analyzing process data and using qualitative content analysis within scoping study methodology requires further discussion.

### Framework stage five: Collating, summarizing, and reporting the results

Stage five is the most extensive in the scoping process, yet it lacks detail in the Arksey and O'Malley framework. Scoping studies have been criticized for rarely providing methodological detail about how results were achieved [1]. We appreciate the importance of breaking the analysis phase into meaningful and systematic steps so that researchers can provide this undertake scoping studies and report on findings in a rigorous manner. As a result, we recommend three distinct steps in framework stage five to increase the consistency with which researchers undertake and report scoping study methodology: analyzing the data, reporting results, and applying meaning to the results. As described in the existing framework, analysis (otherwise referred to as collating and summarizing) should involve a descriptive numerical summary and a thematic analysis. Arksey and O'Malley [6] describe the need to provide a descriptive numerical summary, stating that researchers should describe the characteristics of included studies, such as the overall number of studies included, types of study design, years of publication, types of interventions, characteristics of the study populations, and countries where studies were conducted. However, the description of thematic analysis requires additional detail to assist authors in understanding and completing this step. In our experience, this analytical stage resembled qualitative data analytical techniques, and researchers may consider using qualitative content analytical techniques [10] and qualitative software to facilitate this process.

Second, when reporting results, we recommend that researchers consider the best approach to stating the outcome or end product of the study and how the scoping study findings will be articulated to readers (*e.g*., through themes, a framework, or a table of strengths and gaps in the evidence). This product should be tied to the purpose of the scoping study as recommended in framework stage one.

Finally, in order to advance the legitimacy of scoping study methodology, we must consider the implications of findings within the broader context. As a result, we recommend that researchers consider the meaning of their scoping study results and the broader implications for research, policy, and practice. For example, for the question 'how are motor-learning strategies used within contemporary physical and occupational therapy intervention approaches for children with neuromotor conditions?,' the author (DL) presented themes that described strategy use. Results yielded insights into how researchers should better describe interventions in their publications and provided further considerations for clinicians to make informed decisions about which therapeutic approach might best fit their clients' needs. Considering the overall implications of the results as an explicit framework stage will help to ensure that scoping study results have practical implications for future clinical practice, research, and policy. This recommendation leads to the final stage of the framework.

### Optional stage six: Consultation

Arksey and O'Malley [6] suggest that consultation is an optional stage in conducting a scoping study. Although only one of our three scoping studies incorporated this stage, we argue that it adds methodological rigor and should be considered a required component. Arksey and O'Malley [6] suggest that the purposes of consulting with stakeholders are to offer additional sources of information, perspectives, meaning, and applicability to the scoping study. However, it is unclear when, how, and why to consult with stakeholders, and how to analyze and integrate these data with the findings. We recommend researchers clearly establish a purpose for the consultation, which may include sharing preliminary findings with stakeholders, validating the findings, or informing future research. We suggest researchers use preliminary findings from stage five (either in the form of a framework, themes, or list of findings) as a foundation from which to inform the consultation. This will enable stakeholders to build on the evidence and offer a higher level of meaning, content expertise, and perspective to the preliminary findings. We also recommend that researchers clearly articulate the type of stakeholders with whom they wish to consult, how they will collect the data (*e.g*., focus groups, interviews, surveys), and how these data will be analyzed, reported, and integrated within the overall study outcome.

Finally, given that consultation requires researchers to orient stakeholders on the scoping study purpose, research question, preliminary findings, and plans for dissemination, we recommend that this stage additionally be considered a knowledge transfer mechanism. This may address Brien *et al*.'s [5] concern about the usefulness of scoping studies for stakeholders and how to translate knowledge about scoping studies. Given the importance of knowledge transfer and exchange in the uptake of research evidence [12,13], the consultation stage can be used to specifically translate the preliminary scoping study findings and develop effective dissemination strategies with stakeholders in the field, offering additional value to a scoping study.

One scoping study included a consultation phase comprised of focus groups and interviews with 28 stakeholders including people living with HIV, researchers, educators, clinicians, and policy makers [7]. Authors shared preliminary findings from the literature review phase of the scoping study with stakeholders and asked whether they may be able to identify any additional emerging issues related to HIV and rehabilitation not yet published in the evidence. The team proceeded to conduct a second consultation with 17 new and returning stakeholders whereby the team presented a preliminary framework of HIV and rehabilitation research and stakeholders refined the framework to further identify six key research priorities on HIV and rehabilitation. This series of consultations engaged community members in the development of the study outcome and provided opportunities for knowledge transfer about HIV and rehabilitation research. This process offered an ideal mechanism to enhance the validity of the study outcome while translating findings with the community. Nevertheless, further development of steps for undertaking knowledge translation as a part of the scoping study framework is required.

### Additional considerations for scoping studies to support the advancement, application, and relevance of scoping studies in health research

#### Scoping study terminology

Discrepancies in nomenclature between 'scoping reviews,' 'scoping studies,' 'scoping literature reviews,' and 'scoping exercises' lead to confusion. Despite our collective use of the Arksey and O'Malley framework, two authors (DL, HC) titled their studies as 'scoping reviews' while the other used 'scoping study.' In this paper, we use 'scoping studies' for consistency with Arksey and O'Malley's original framework. Nevertheless, the potential differences (if any) among the terms merit clarification. Lack of a universal definition for scoping studies is also problematic to researchers trying to clearly articulate their reasons for undertaking a scoping study. Finally, we advocate for labeling the methodology as the 'Arksey and O'Malley framework' to provide consistency for future use.

#### Quality assessment

Another consideration for scoping study methodology is the potential need to assess included studies for methodological quality. Brien *et al. *[5] state that this lack of quality assessment makes the results of scoping studies more challenging to interpret. Grant and Booth [4] imply that a lack of quality assessment limits the uptake of scoping study findings into policy and practice. While our research questions did not directly relate to any quality assessment debate, we recognize the challenges in assessing quality among the vast range of published and grey literature that may be included in scoping studies. This also raises the question of whether and how evidence from stakeholder consultation is evaluated in the scoping study process. It remains unclear whether the lack of quality assessment impacts the uptake and relevance of scoping study findings.

A final consideration for legitimization of scoping study methodology includes the development of a critical appraisal tool for scoping study quality [5]. Anderson *et al. *[2] offer criteria for assessing the value and utility of a commissioned scoping study in health policy contexts, but these criteria are not necessarily applicable to scoping studies in other areas of health research. Developing a critical appraisal tool would require the elements of a methodologically rigorous scoping study to be defined. This could include, but would not be limited to, the minimum level of analysis required and the requirements for reporting results. Overall, the issues surrounding quality assessment of included studies and subsequent scoping studies require further discussion.

### Limitations

This paper responds to Arksey and O'Malley's [6] request for feedback to their proposed methodological framework. However, the recommendations that we propose are derived from our subjective experiences undertaking scoping studies of varying sizes in the rehabilitation field, and we recognize that they may not represent the opinions of all scoping study authors. Other than our individual experiences with our own studies, we have not yet implemented the full framework recommendations. Hence, readers can determine how strongly to interpret and implement these recommendations in their scoping study research. We invite others to trial our recommendations and continue the process of refining and improving this methodology.

## Summary

Scoping studies present an increasingly popular option for synthesizing health evidence. Brien *et al. *[5] argue that guidelines are required to facilitate scoping review reporting and transparency. In this paper, we build on the existing methodological framework for scoping studies outlined by Arksey and O'Malley [6] and provide recommendations to clarify and enhance each stage, which may increase the consistency with which researchers undertake and report scoping studies. Recommendations include: clarifying and linking the purpose and research question; balancing feasibility with breadth and comprehensiveness of the scoping process; using an iterative team approach to selecting studies and extracting data; incorporating a numerical summary and qualitative thematic analysis; identifying the implications of the study findings for policy, practice, or research; and adopting consultation as a required component of scoping study methodology. Ongoing considerations include: establishing a common accepted definition and purpose(s) of scoping studies; defining methodological rigor for the assessment of scoping study quality; debating the need for quality assessment of included studies; and formalizing knowledge translation as a required element of scoping methodology. Continued debate and development about scoping study methodology will help to maximize the usefulness of scoping study findings within healthcare research and practice.

## Competing interests

The authors declare that they have no competing interests.

## Authors' contributions

DL and HC conceived of this paper. DL undertook the literature review process. DL, HC and KO developed challenges and recommendations. All authors drafted the manuscript. All authors read and approved the final manuscript.

## Authors' information

DL is a physical therapist and doctoral candidate in the School of Rehabilitation Science at McMaster University. HC is an occupational therapist and doctoral candidate in the School of Rehabilitation Science at McMaster University. KO is a clinical epidemiologist, physical therapist, and postdoctoral fellow in the School of Rehabilitation Science at McMaster University. She is also a Lecturer in the Department of Physical Therapy at the University of Toronto.
